# Integrated Proteomic Profiling Reveals Dynamic Remodeling of the Intestinal Proteome in *Toxoplasma gondii*-Infected C57BL/6J Mice

**DOI:** 10.3390/biology15171461

**Published:** 2026-08-27

**Authors:** Zhi-Lin Li, Yu-Xin Zhang, Pei-Lin Wang, Chen Liu, Nan Chen, Feng-Cai Zou, Xing-Quan Zhu, Zhao Li

**Affiliations:** 1 Key Laboratory of Medicinal Chemistry for Natural Resource, Ministry of Education, Yunnan Key Laboratory of Research and Development for Natural Products, School of Pharmacy, Yunnan University, Kunming 650500, China; lizhilin@ynu.edu.cn (Z.-L.L.); yuxin_zhang2025@163.com (Y.-X.Z.); 2 Animal Research and Resource Center, School of Life Sciences, Yunnan University, Kunming 650201, China; peilinwang2026@163.com (P.-L.W.); tim633511@gmail.com (C.L.); c2179229244@163.com (N.C.); 3 The Yunnan Key Laboratory of Veterinary Etiological Biology, College of Veterinary Medicine, Yunnan Agricultural University, Kunming 650201, China; zfc1207@vip.163.com (F.-C.Z.); xingquanzhu1@hotmail.com (X.-Q.Z.); 4 Shanxi Key Laboratory of Animal Disease Research, College of Veterinary Medicine, Shanxi Agricultural University, Jinzhong 030801, China

**Keywords:** *Toxoplasma gondii*, intestinal proteome, host–pathogen interaction, resource reallocation, ribosome biogenesis

## Abstract

The intestinal mucosa is the primary site of *Toxoplasma gondii* (*T. gondii*) infection and interaction with the host. While the intestinal responses have been characterized through transcriptomic and histopathological studies, a comprehensive, system-wide analysis of the functional proteome remodeling by acute *T. gondii* infection is required to elucidate the underlying mechanisms. This study presents a temporal, quantitative proteomic profiling of *T. gondii*-infected C57BL/6J mouse intestine to define the protein-centric host response. Global analysis reveals profound reprogramming, characterized by upregulated acute-phase reactants and interferon-stimulated effectors and downregulated epithelial barrier and digestive function proteins. Systems-level bioinformatics analysis uncovers a coordinated host strategy of cellular resource reallocation, evidenced by the simultaneous and specific amplification of ribosome biogenesis and proteasomal degradation pathways. Subsequent protein–protein interaction network analysis substantiates this strategic investment in core protein homeostasis infrastructure, identifying the ribosome biogenesis machinery as the topological core of the response interactome. These findings support a model of defense-priority resource reallocation. That is, the host redirects cellular resources to optimize its biosynthetic and catabolic capacity, facilitating a high-output immune response at the potential expense of tissue homeostasis. This work provides an integrated proteomic atlas of the intestinal immunopathology mechanisms during toxoplasmosis and establishes a foundation for future host-directed therapeutic exploration.

## Simple Summary

*Toxoplasma gondii* is a common parasite that infects the intestines of a wide range of animals, including humans, yet we still do not fully understand how the intestine responds to this invader at the level of proteins, i.e., the actual molecules undertaking most intracellular functions. In this study, we infected mice with this parasite and measured thousands of proteins in their intestinal tissues with a high-sensitivity technology. The results show that the parasite causes extensive protein landscape reorganization in the gut. The host activates numerous proteins involved in inflammation and anti-parasite defense while reducing proteins that maintain normal digestion and the protective gut barrier. More importantly, we discovered that the host strategically boosts its cellular machinery for generating and recycling proteins, likely to meet the high demand for defensive proteins. These findings point to a trade-off: the gut prioritizes anti-infection by directing resources away from routine functions, possibly contributing to tissue damage during the acute infection. This study plots a comprehensive protein map that offers new insights into gut defense mechanisms and may help guide future efforts to develop therapies that modulate the host response rather than targeting the parasite directly.

## 1. Introduction

*Toxoplasma gondii* (*T. gondii*) is an obligate intracellular apicomplexan parasite that infects a broad spectrum of warm-blooded animals, affecting approximately one-third of the global human population [1,2]. While *T. gondii* infection typically remains asymptomatic in immunocompetent individuals, congenital transmission or infection in immunocompromised patients can lead to encephalitis, chorioretinitis, and other life-threatening complications [3]. Beyond the immediate threats of acute pathogenesis, chronic *T. gondii* infection with persistent tissue cysts predominantly in the central nervous system and musculature has been epidemiologically linked to subtle neuropsychiatric disorders [4]. The biological complexity of *T. gondii* is underscored by its multi-stage life cycle. The sexually reproductive stage occurs exclusively in the feline intestine, generating oocysts shed into the environment. Upon ingestion by intermediate hosts, including rodents and humans, the parasite undergoes rapid expansion as tachyzoites and subsequently differentiates into long-lived bradyzoites within cysts [5,6]. Following oral infection, the host intestinal mucosa becomes the primary site for the invading sporozoites or bradyzoites to transform into tachyzoites, thereby initiating a critical enteric phase that governs the extent of systemic dissemination and the establishment of chronic infection [7]. Therefore, host–parasite interactions at the intestinal interface are fundamental to elucidating the pathogenesis of toxoplasmosis.

The intestinal response to *T. gondii* infection is highly orchestrated and dynamic, involving intricate crosstalk among the mucosal immune system, the epithelial barrier, and the resident commensal microbiota [8]. A robust Type 1 immune response is initiated following infection, dominated by IFN-γ production, which is critical for controlling parasite replication while also contributing to immunopathology [9]. The activation of intraepithelial lymphocytes, lamina propria dendritic cells, and macrophages is involved in this response [10]. Concurrently, infection disrupts intestinal barrier integrity, manifested as the altered expression and localization of tight junction proteins (e.g., occludin, claudins) and increased permeability [11]. Furthermore, emerging evidence highlights bidirectional interactions between *T. gondii* and the gut microbiome [12,13]. Infection induces significant dysbiosis, characterized by microbial community structure variations, while the microbiota composition, in turn, modulates host susceptibility to infection and the ensuing inflammatory response [14,15]. Nonetheless, existing knowledge largely stems from transcriptomic or histopathological analyses, which primarily offer genetic or morphological insights. A comprehensive, protein-level description of the intestinal proteome’s remodeling post-infection remains incomplete.

Transcriptomics has proven invaluable in mapping gene expression changes. However, mRNA abundance is not always correlated with functional protein levels due to post-transcriptional regulation, translational control, and protein degradation. High-throughput proteomics, particularly liquid chromatography-tandem mass spectrometry (LC-MS/MS), offers a powerful complementary approach that is often more functionally relevant [16]. It facilitates global quantification of protein expression, identification of post-translational modifications (e.g., phosphorylation and acetylation), and revelation of activated pathways directly linked to cellular phenotype. Regarding host–pathogen interactions, proteomics can uncover novel host defense proteins, detail the parasite-mediated manipulation of host signaling networks, and shed light on metabolic adaptation details that are frequently obscured in transcriptomic data alone [17,18,19].

Given the pivotal role of the intestine in toxoplasmosis pathogenesis and the transformative potential of proteomics, we hypothesize that acute *T. gondii* infection profoundly and specifically remodels the host intestinal proteome, and that proteomics can reveal novel pathways involved in intestinal barrier dysfunction, immune activation, and metabolic adaptation that are not fully captured at the transcriptional level. To test these hypotheses, this study (1) systematically characterizes the murine intestinal proteome at day 10 post-infection using quantitative LC-MS/MS; (2) performs comprehensive functional annotation of dysregulated proteins and pathways through KEGG, GO, and PPI network analyses; and (3) discusses the biological relevance of the identified proteomic changes in the context of existing transcriptomic knowledge, thereby providing a protein-centric resource that complements and extends current understanding of intestinal toxoplasmosis. The findings will provide a crucial protein-centric resource and advance our mechanistic understanding of intestinal immunopathology during *T. gondii* infection.

## 2. Methods and Materials

### 2.1. Ethical Approval and Experimental Animals

All animal experiments were conducted in strict accordance with the Guidelines for the Management and Use of Laboratory Animals established by the Animal Research and Resource Center of Yunnan University (CNAS LA0029). The animal use protocol and ethics were reviewed and approved by the Institutional Animal Care and Use Committee of Yunnan University (Approval No. YNU20241024; date: 2 February 2024).

Laboratory Animal Center of Yunnan University (Kunming, China) supplied 6- to 8-week-old specific-pathogen-free (SPF) female C57BL/6J mice. All mice were housed in an ABSL-2 facility maintained at 22 ± 2 °C and a 12 h light/dark cycle, with ad libitum access to sterile feed and water. Prior to experimentation, all mice were confirmed to be seronegative for *T. gondii* by ELISA.

### 2.2. Parasite Preparation and Cyst Isolation

The *T. gondii* ME49 strain (Type II) was maintained in our laboratory by serial passage in ICR mice. Tissue cysts for the infection experiments were extracted from brains harvested from chronically infected mice (≥30 days post-infection). Briefly, in summary, the brain tissues of ICR mice were homogenized in sterile phosphate-buffered saline (PBS). The number of *T. gondii* cysts was counted using a hemocytometer under a microscope. The cyst suspension was diluted with PBS to a final concentration of 1000 cysts/mL and immediately used for an oral infection experiment in C57BL/6J mice [20].

### 2.3. Experimental Design and Sample Collection

The C57BL/6J mice were randomly divided into two groups (*n* = 3 each): the *T. gondii*-infected group (CJA) and the uninfected control group (CJN). Mice in the CJA group were orally gavaged with 100 μL of PBS containing 100 ME49 tissue cysts to simulate natural infection, while CJN mice received an equivalent volume of sterile PBS.

At 10 days post-infection, all mice were euthanized by CO_2_ inhalation. Following dissection, a colon tissue segment (approximately 1.5 cm in length) was quickly collected from each mouse. After removing luminal contents by gently flushing with ice-cold PBS, the intestinal segments were snap-frozen in liquid nitrogen and stored at −80 °C until further analysis.

To confirm infection, genomic DNA was extracted from all collected tissues using the Tiangen Genomic DNA Kit (Tiangen, Beijing, China) following the manufacturer’s instructions. The *T. gondii B1* gene was amplified using a previously described semi-nested PCR method and visualized by electrophoresis using a 2.0% agarose gel.

### 2.4. Protein Extraction, Quality Control, and Digestion

Proteomic analysis was commissioned to BGI Genomics Co., Ltd. (Shenzhen, China). The detailed workflow was as follows.

In the differential proteomic analysis, three biological replicates (*n* = 3) per group were employed, with each replicate originating from a separate mouse. Approximately 20 mg of frozen colon tissue was homogenized in a lysis buffer (7 mol/L urea, 2 mol/L thiourea, 20 mmol/LM Tris–HCl (pH = 8.5), 0.2% SDS, and protease inhibitor cocktail) on ice using a tissue grinder. The homogenate was centrifuged at 25,000× *g* for 15 min at 4 °C, and the supernatant was collected. The protein concentration was measured by Bradford assay. Protein integrity was checked by loading a 10 μg sample onto a 12% SDS-PAGE gel.

The protein solution (100 μg) was digested in a 10 kDa ultrafiltration tube. The proteins were first reduced with 10 mmol/L dithiothreitol (DTT) at 56 °C for 1 h and then alkylated with 55 mmol/L iodoacetamide (IAM) in the dark at room temperature for 45 min. Trypsin was added at an enzyme-to-substrate ratio of 1:20 (*w*/*w*), and the mixture was incubated at 37 °C for 4 h. The resulting peptide solution was desalted using Strata X columns and then vacuum-dried and stored at −80 °C until LC-MS/MS analysis.

### 2.5. Liquid Chromatography-Tandem Mass Spectrometry

Dried peptide samples were reconstituted in mobile phase A (2% acetonitrile, 0.1% formic acid) and centrifuged at 20,000× *g* for 10 min. The supernatant was injected into a Thermo Scientific Vanquish Neo UHPLC system (Munich, Germany) with an Orbitrap Astral mass spectrometer. Peptides were separated on an EASY-Spray^TM^ HPLC column (150 μm × 15 cm, Thermo Scientific, Waltham, MA, USA) at a flow rate of 0.8 to 2.5 μL/min using the following effective gradient: 0 to 0.5 min, mobile phase B (80% acetonitrile, 0.1% formic acid) increased from 4% to 5% with flow rate decreasing from 2.5 to 2 μL/min; 0.5 to 0.9 min, B increased from 5% to 8.5% with flow rate decreasing to 0.8 μL/min; 0.9 to 14.2 min, B increased from 8.5% to 25%; 14.2 to 21.1 min, B increased from 25% to 35%; 21.1 to 21.5 min, B increased from 35% to 55% with flow rate increasing to 2 μL/min; 21.5 to 21.9 min, B increased from 55% to 99% with flow rate increasing to 2.5 μL/min; 21.9 to 22.7 min, 99% B was maintained.

Mass spectrometric detection was performed in the Data-Independent Acquisition (DIA) mode. The instrument was equipped with a nanoESI source operating at an ion spray voltage of 1.8 kV. Precursor (MS1) spectra were acquired over the range of 380 to 980 *m*/*z* at a resolution of 240,000 with a maximum injection time (MIT) of 5 ms. The 380 to 980 *m*/*z* range was divided into 300 variable isolation windows for sequential windowed fragmentation and signal acquisition. Peptide fragmentation was achieved via higher-energy collisional dissociation (HCD) with a normalized collision energy (NCE) of 25%. Fragment ions were detected in the Astral mass analyzer with an MIT of 3 ms. Automatic gain control (AGC) targets were set to 500% for both MS1 and MS2.

### 2.6. Database Search, Protein Identification, and Quantification

A project-specific spectral library was constructed by first fractionating and analyzing the pooled peptide samples in the Data-Dependent Acquisition (DDA) mode. Raw DDA data were processed in MaxQuant (version 1.5.3.30) using a built-in Andromeda search engine against a concatenated UniProt database containing protein sequences from *Mus musculus* and *T. gondii*. A false discovery rate (FDR) of 1% was strictly applied at both the peptide and protein levels.

Based on the constructed spectral library, raw DIA data were subjected to peak extraction and retention time alignment. Proteins were identified and quantified using dedicated algorithms, and the relative protein abundance was calculated based on the integrated peak areas of fragment ions from constituent peptides.

### 2.7. Quality Control and Statistical Analysis

During data quality assessment, (1) intra-group coefficient of variation (CV) analysis was performed to evaluate quantitative reproducibility; (2) principal component analysis (PCA) and non-metric multi-dimensional scaling (NMDS) were conducted to examine overall sample distribution; and (3) the Pearson correlation heatmap of protein expression across samples was plotted to verify consistency among biological replicates.

Differentially expressed proteins (DEPs) between the CJA and CJN groups were identified using Welch’s two-tailed *t*-test. Proteins with |Fold Change| ≥ 2 and *p* < 0.001 were considered significantly differentially expressed. False discoveries arising from multiple hypothesis testing were controlled by adjusting the *p*-values using the Benjamini-Hochberg procedure, and corresponding q*-values were calculated.

### 2.8. Bioinformatics Analysis

Systematic functional annotation and enrichment analyses were conducted on the entire set of identified proteins and the subset of significantly differentially expressed proteins. The specific analyses included: (1) gene ontology (GO) annotation and enrichment analysis, covering the three ontologies of Biological Process (BP), Cellular Component (CC), and Molecular Function (MF); (2) Kyoto Encyclopedia of Genes and Genomes (KEGG) pathway annotation and enrichment analysis; and (3) protein–protein interaction (PPI) network construction for DEPs. All annotation and enrichment analyses were performed on the Dr. Tom platform (BGI Genomics, Shenzhen, China, accessed on 9 February 2020). Across all enrichment analyses, the background set comprised all identified proteins, and statistical significance was determined using the hypergeometric test, with *p* < 0.05 considered significantly enriched. The complete list of identified proteins with peptide counts, sequence coverage, and other identification parameters is provided in Supplementary Table S1. The full dataset of differentially expressed proteins, including log_2_ fold-change values, *p*-values, and Q-values, is available in Supplementary Table S2.

## 3. Results and Discussion

### 3.1. Global Proteomic Profiling Reveals Profoundly Remodeled Intestinal Proteome upon Infection

**Figure 1:**
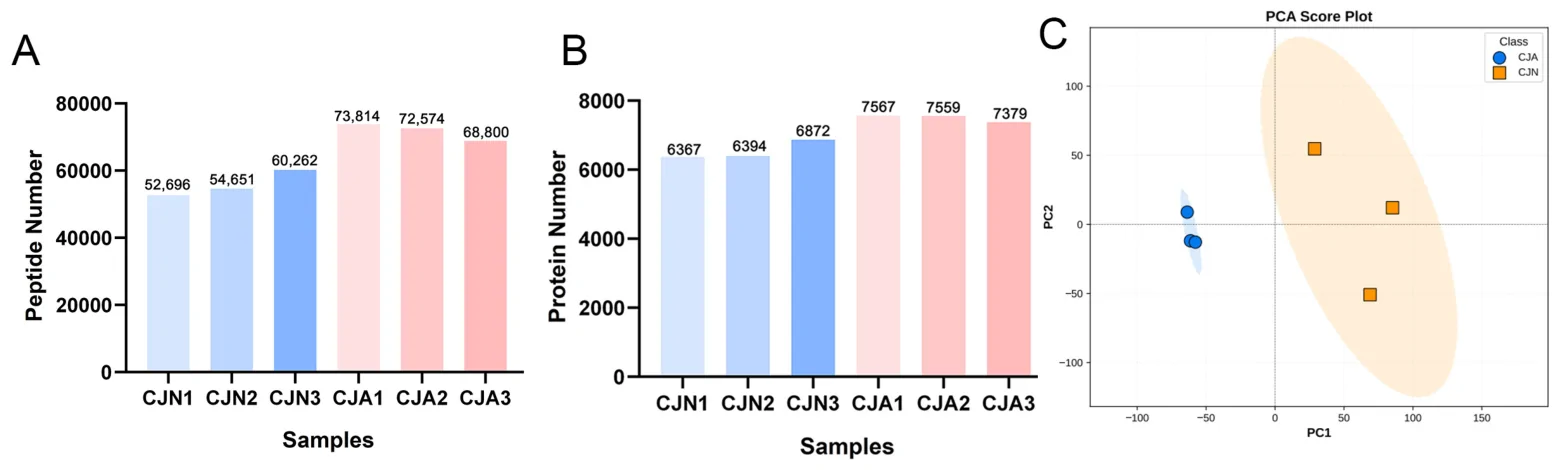
Global proteomic profiling reveals distinct intestinal proteome remodeling upon *T. gondii* infection. (**A**). Number of unique peptides identified in CJN and CJA intestinal tissues (*n* = 3 biological replicates per group). (**B**). Total number of quantified proteins in CJN and CJA groups. (**C**). PCA score plot of the proteomic profiles. Infected samples (blue) are distinctly separated from controls (orange-yellow) along the first principal component (PC1, 60.64% variance).

A comprehensive, protein-centric view of the host intestinal response to acute *T. gondii* infection was established using label-free quantitative proteomic analysis on whole intestinal tissues from C57BL/6J mice in the CJA and CJN groups (*n* = 3 biologically independent replicates per group). High-resolution LC-MS/MS data were processed in MaxQuant, with stringent filters applied to ensure high-confidence identification (FDR < 1% for both peptide and protein). Initial quality metrics revealed a significant increase in proteomic identifications in infected samples. The number of unique peptides identified per replicate was 71,729 ± 2611 in the CJA group, more than that in the CJN group (55,869 ± 3927; Figure 1A and Table S1). Correspondingly, the total number of quantified proteins was 7501 ± 106 in CJA samples versus 6544 ± 284 in controls (Figure 1B). These consistent increments in the identifications may be partly attributed to the altered tissue cellularity and protein abundance induced by the infection. Acute *T. gondii* infection is known to trigger extensive immune cell infiltration (e.g., lymphocytes, neutrophils, and monocytes) and profoundly alters the composition and function of intestinal epithelial cells, which could collectively contribute to an expanded protein pool in the infected tissues. Additionally, infection-induced tissue edema and epithelial cell shedding may change the relative abundance of extracellular matrix and cytoskeletal proteins, further diversifying the detectable proteome. However, since our data were not normalized to total cell number or tissue mass, this interpretation remains a hypothesis until further experimental validation. Alternative explanations, such as infection-enhanced protein extraction efficiency or differential protein solubility due to tissue damage, cannot be formally excluded. Regardless of the underlying driver, the consistent increase in identifications across all infected replicates supports a genuine biological difference rather than technical variability, and the distinct separation observed in the PCA findings provides confidence that the downstream functional analysis captures infection-related alterations. Equally important, the observed proteomic signatures represent averages across all cell types present in the whole-tissue lysates. Thus, the enriched pathways such as ribosome biogenesis and proteasomal degradation may partly reflect the increased representation of infiltrating immune cells rather than cell-autonomous reprogramming of epithelial cells. Consequently, the “resource reallocation” framework discussed below should be interpreted as a correlative model consistent with our observations, rather than a definitive causal mechanism.

The overall reproducibility of the constructed proteomic dataset and the infection-induced variance were assessed via unsupervised PCA. The PCA score plot showed clear, pronounced segregation between the CJA and CJN groups along PC1, which accounted for 60.64% of the total variance (Figure 1C). The second component (PC2, 15.45% of total variance) primarily captured intra-group variability. Critically, the tightly clustered biological replicates under each condition (average intra-group correlation > 0.92) confirmed the high technical reproducibility of the sample preparation and analysis processes herein. The distinct separation along PC1 (inter-group correlation < 0.75) provided robust statistical evidence that oral *T. gondii* infection induced a major coordinated transformation of the host intestinal proteomic landscape. This global shift served as a molecular signature of the intense multifaceted host–pathogen dialogue at the primary site of parasite invasion, enabling the thorough exploration of the specific pathways involved.

### 3.2. Identification of Differentially Expressed Proteins Highlights a Dominant Anabolic Immune Response and Barrier Compromise

**Figure 2:**
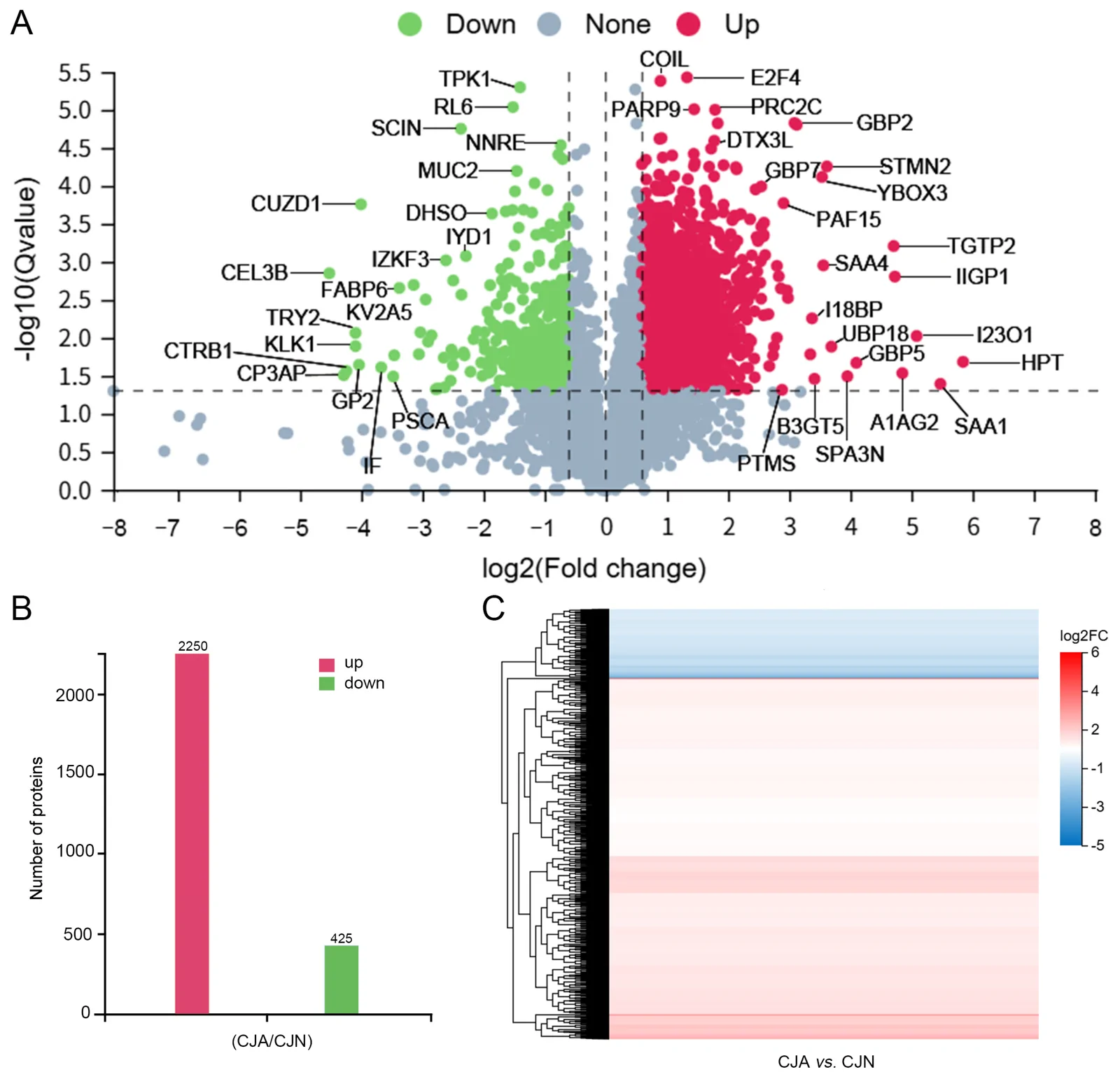
Identification and functional overview of the intestinal differentially expressed proteome. (**A**) Volcano plot of protein abundance changes. Each point represents a quantified protein. Significantly upregulated and downregulated proteins are highlighted in red and green, respectively. Key markers of acute inflammation (e.g., SAA1), anti-parasitic defense (e.g., IRGM1), and barrier integrity (e.g., SCIN) are annotated. (**B**) Quantification of DEPs. The bar graph shows the total numbers of significantly upregulated (2250) and downregulated (425) proteins. (**C**) Hierarchical clustering of DEPs. Heatmap displaying log_2_FC values for all DEPs across various conditions. Rows (proteins) are clustered by expression pattern, visually presenting the dominant upregulation profile and revealing co-regulated protein groups.

Proteins showing significant changes in abundance were identified via differential expression analysis using a two-step threshold: an absolute log_2_ fold-change |log_2_FC| > 1 (corresponding to a >2-fold change) and an adjusted *p*-value (Q-value) < 0.001. This stringent approach yielded 2675 DEPs, with 2250 (84.1%) significantly upregulated and only 425 (15.9%) downregulated in infected intestines (Figure 2B, the complete DEP dataset is provided in Supplementary Table S2). This overwhelming bias toward upregulation underscores the heavy reliance of the host’s frontline defense strategy on the de novo synthesis and deployment of a vast array of effector molecules, rather than the selective suppression of specific pathways.

The volcano plot provides a statistically rigorous overview of the profound proteomic rewiring following acute *T. gondii* infection (Figure 2A). Among the most significantly upregulated proteins, the dramatic induction of Serum Amyloid A-1 (SAA1, log_2_FC = 5.47) and Haptoglobin (HPT, log_2_FC = 5.84) stands out. Instead of being merely systemic acute-phase reactants, their robust localized expression in intestinal tissue signifies a severe, IL-1/IL-6-driven mucosal inflammatory storm capable of altering endothelial and epithelial permeability, potentially facilitating parasite dissemination while mobilizing innate defenses [21,22]. This inflammatory context is inextricably linked to a dominant IFN-γ-driven transcriptional program, as evidenced by the coordinated upregulation of a suite of interferon-stimulated genes (ISGs). Central to this are members of the guanylate-binding protein (GBP) family [23]. An orchestrated upregulation of GBP2 (log_2_FC = 3.12), GBP5 (log_2_FC = 4.10), and GBP7 (log_2_FC = 2.54) was observed. This is of particular functional significance, as GBPs oligomerize and form pores on the parasitophorous vacuole membrane (PVM), eventually leading to its disintegration, a critical cell-autonomous defense mechanism against *T. gondii* [24,25]. The multiple GBP isoforms suggest a robust, non-redundant assault on the parasite’s intracellular niche. Previous research has confirmed Gbp1 and mGBP2 as induced by IFN-γ, which contribute to host resistance against *T. gondii*. IFN-γ-activated macrophages recruit Gbp1 to the parasitophorous vacuole, which is associated with membrane vesiculation, vacuolar disruption, and subsequent parasite clearance. The less efficient elimination of susceptible parasites by Gbp1-deficient macrophages further supports its role in cell-autonomous immunity [25,26]. Parasite virulence factors also affect this response, as ROP5 and ROP18 can restrain Gbp1 recruitment to the parasitophorous vacuole, allowing the parasite to evade the above host defense mechanism [27]. Meanwhile, mGBP2 plays a related but distinct role by accumulating around the parasitophorous vacuole and restricting intracellular parasite replication. Loss of mGBP2 results in impaired parasite control and increased host susceptibility. Previous literature also showed that mGBP2 expression is strongly induced by IFN-γ and markedly reduced in IRF1-deficient cells, indicating IRF1’s contribution to its transcriptional regulation. Nonetheless, mGBP2 is also induced by type I interferons, TNF, and Toll-like receptor agonists, suggesting that several overlapping inflammatory signals shape GBP expression [25,26]. The simultaneous increases in GBP2, GBP5, and GBP7 align with the activation of an interferon-related, cell-autonomous anti-parasitic response in the infected intestine [27]. Without direct measurement of IFN-γ levels and IRF1 activity, however, the findings herein should be regarded as supportive evidence rather than direct confirmation of an IFN-γ-IRF1-GBP regulatory pathway. Furthermore, the significant upregulation of Poly (ADP-ribose) polymerase 9 (PARP9, log_2_FC = 1.45) alongside its binding partner Deltex E3 Ubiquitin Ligase 3L (DTX3L, log_2_FC = 1.45) points to a layer of signal amplification and epigenetic regulation during IFN response. The PARP9-DTX3L complex is well-established to enhance type I and II IFN signaling by upregulating an ISG subset, potentially creating a positive feedback loop that sustains the anti-parasitic state [28].

Conversely, the downregulated proteome indicates collateral damage and strategic host reprogramming. Scinderin (SCIN, log_2_FC = −2.37) was also markedly downregulated. While SCIN has been primarily characterized for its role in actin cytoskeleton remodeling in other cellular contexts, its downregulation in the intestinal epithelium suggests a potential role in maintaining intestinal barrier integrity, though further confirmation is required [29]. Its depletion likely contributes to the increased intestinal permeability during toxoplasmosis. Beyond structural components, the functional components of intestinal homeostasis are also downregulated. The reduction of Mucin 2 (MUC2, log_2_FC = −1.46), the core protein of the protective mucus layer, suggests chemical barrier erosion, potentially exposing the epithelium to the parasite and commensal bacteria [18]. Simultaneously, a broad suppression of key digestive enzymes (e.g., trypsinogens, chymotrypsin-like elastase, and carboxypeptidases) and nutrient transporters indicates a potential global dampening of digestive and absorptive functions. This, coupled with the downregulation of metabolic enzymes (e.g., Thymidine Phosphorylase 1, TPK1, log_2_FC = −1.40), supports an anabolic repression model of the host. Collectively, this downregulation pattern aligned with a potential host-mediated resource-allocation strategy, with energy and biosynthetic precursors diverted from routine maintenance and digestion toward the energetically demanding processes of immune effector production. It may alternatively reflect a parasite-driven host environment manipulation that enhances nutrient availability for replication or constitute a direct outcome of infection-induced epithelial damage and turnover [30].

### 3.3. Systems-Level Functional Analysis of Host Response

#### 3.3.1. KEGG Pathway Enrichment Reveals Co-Activation of Immune Defense, Proteostasis, and Biosynthetic Programs

**Figure 3:**
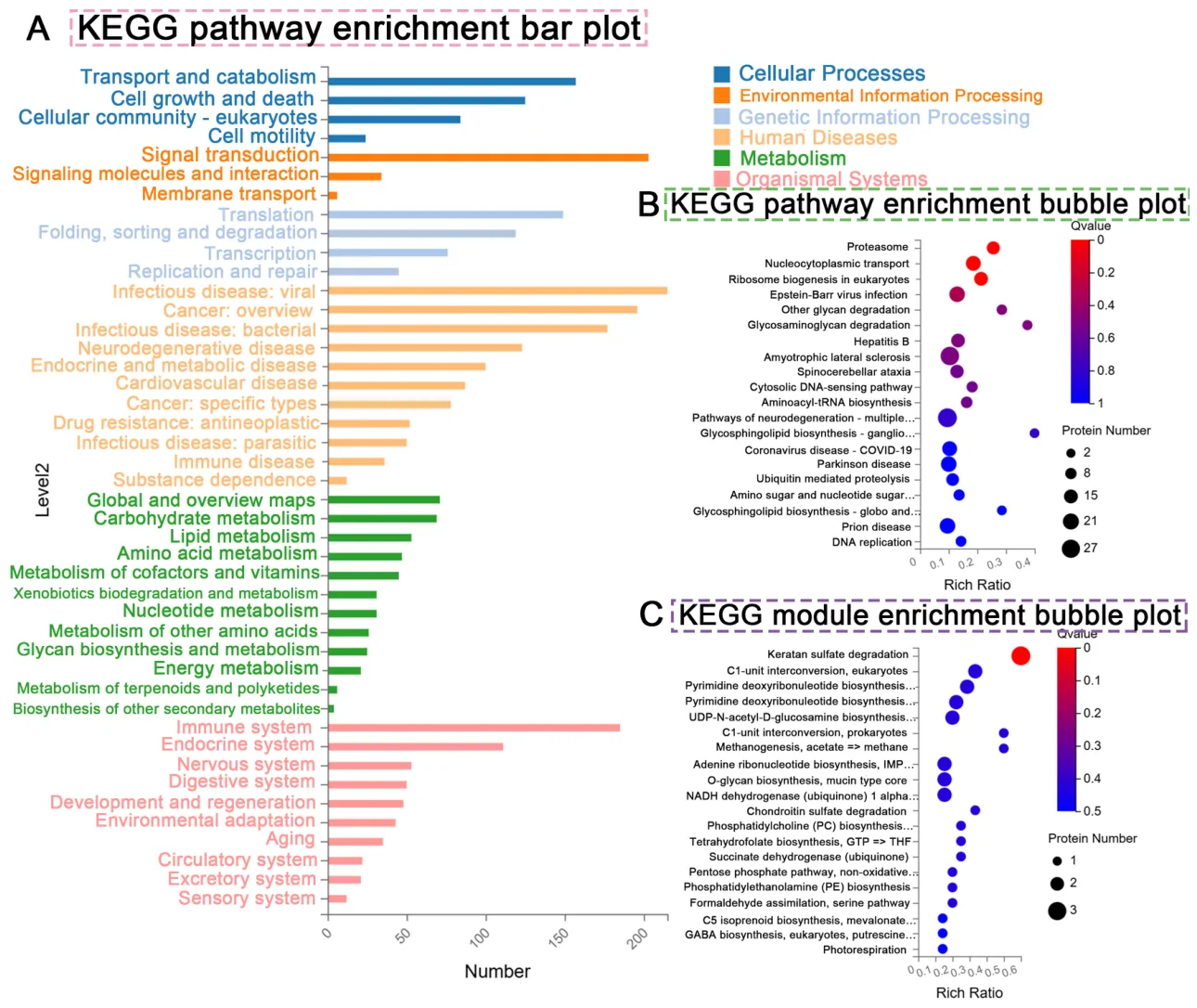
KEGG pathway and module enrichment analysis of the intestinal proteomic response to *T. gondii* infection. (**A**) Functional distribution of DEPs across KEGG categories. Bar plot shows the number of DEPs mapped to Level 2 pathways, grouped by Level 1 categories (color-coded). (**B**) Enriched KEGG pathways. Bubble plot displays pathway enrichment, with bubble size indicating DEP count and bubble color representing −log_10_(Q-value). Significantly enriched pathways (e.g., Proteasome and Ribosome biogenesis) are labeled. (**C**). Enriched KEGG modules. Bubble plot of module enrichment highlights key modules, such as Keratan sulfate degradation and Pyrimidine deoxyribonucleotide biosynthesis.

Systems-level organization of the host response was revealed through KEGG pathway enrichment analysis, thereby moving beyond the scope of individual DEPs. The results revealed the coherent response of the tissue under severe immune stress (Figure 3A). The proteasome pathway (ko03050) was most significantly enriched. This finding transcended beyond the simple notion of increased protein turnover. The frequent immunoproteasome induction by IFN-γ during *T. gondii* infection enhances peptide antigen generation for MHC-I presentation, which is critical for CD8^+^ T cell-mediated parasite control. Furthermore, the proteasome is essential for the precise and rapid degradation of key signaling molecules (e.g., IκB and cell cycle regulators), allowing dynamic immune and stress responses. Its enrichment highlights the critical need for regulated protein degradation to shape an effective anti-parasitic environment [31]. Concomitantly, the Nucleocytoplasmic Transport pathway (ko03013) was highly enriched, reflecting the intense signaling traffic required for effective innate immune response. Following phosphorylation, transcription factors (e.g., STATs and IRFs) must rapidly translocate to the nucleus to drive ISG expression. Its enrichment reflects the logistical scale of the transcriptional reprogramming within infected intestinal cells.

The significant enrichment of Ribosome biogenesis in eukaryotes (ko03008) was equally crucial. Thus, the host was not merely upregulating specific immune effectors but fundamentally augmenting its core protein synthesis machinery. This finding represented the host’s high-level strategic investment in its ribosomal factory capacity to sustain the massive production of cytokines, chemokines, antimicrobial peptides, and other defense proteins [32]. This finding aligns with the observed downregulation of some structural ribosomal proteins (possibly indicating remodeling) and the upregulation of numerous ribosome biogenesis factors among the identified DEPs. Moderately enriched pathways included the Cytosolic DNA-sensing pathway (ko04623) (Figure 3B), with the former being consistent with the established role of cGAS-STING signaling in sensing *T. gondii* genomic DNA and fueling type I IFN production and the latter relating to inflammasome activation [33].

KEGG module analysis further refined the above finding (Figure 3C). The most strikingly enriched module was Keratan sulfate degradation (M00078). Although Keratan sulfate was a minor component in the intestine, its enrichment likely indicates broader activation of lysosomal and glycosaminoglycan (GAG) catabolism pathways, possibly associated with immune cell extravasation, cytokine/growth factor regulation, or tissue remodeling following damage. Enrichment of modules such as Pyrimidine deoxyribonucleotide biosynthesis (M00048) supported the increased demand for nucleotides necessary for immune cell proliferation and transcriptional activity.

#### 3.3.2. GO Enrichment Delineates the Cellular and Molecular Architecture of Host Response

The proteomic alterations were deconstructed into a coherent functional framework through integrated GO enrichment analysis across the three standard ontologies: BP, CC, and MF. This analysis corroborated the broad pathways identified by KEGG while plotting a higher-resolution, multi-dimensional blueprint of the host’s defensive reprogramming, which detailed the processes mobilized, their spatial organization, and the precise molecular tools employed.

**Figure 4:**
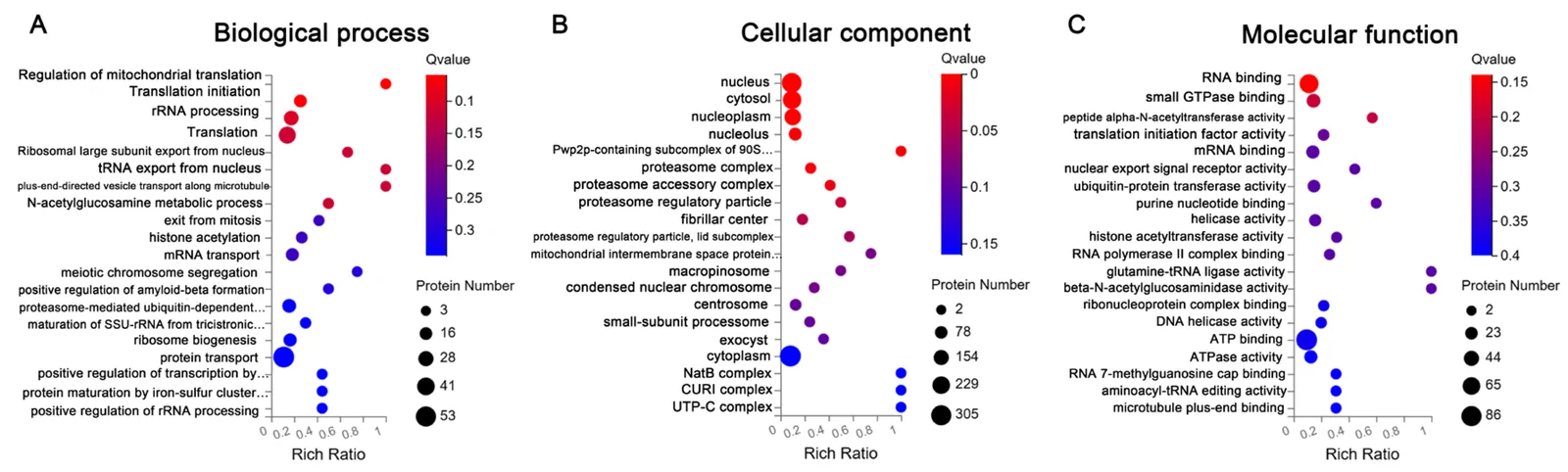
GO enrichment analysis of the intestinal proteomic response to *T. gondii* infection. (**A**) Enriched BP terms. Bubble plot shows the significantly enriched BP terms. (**B**) Enriched CC terms. (**C**) Enriched MF terms. (Bubble size indicates the number of DEPs, and bubble color represents −log_10_(Q-value).

BP analysis revealed a host strategy outlining anabolic surge and catabolic control. It delineated a program fundamentally centered on the extensive upscaling of protein production, as supported by enhanced quality control and turnover (Figure 4A). This was manifested as a coherent anabolic cascade from the positive transcription regulation by RNA polymerase I (GO:0000455) that fuels ribosomal RNA synthesis to key steps of ribosome biogenesis (GO:0042254), including maturation of SSU-rRNA (GO:0000470) and its positive regulation (GO:0043623). This cascade culminated in the strengthening of translational initiation (GO:0006413) and translation (GO:0006412), ensuring both the supply of ribosomal parts and their efficient assembly and deployment. This biosynthetic surge was logistically supported by parallel enrichments in tRNA export from the nucleus (GO:0006409) and mRNA transport (GO:0050658), guaranteeing a steady flow of essential substrates. Notably, the enrichment for regulation of mitochondrial translation (GO:0070129) suggested a coordinated expansion of organellar protein synthesis, likely supporting the biogenesis of oxidative phosphorylation complexes for the heightened energy demand. Concurrently, a robust catabolic program was activated, marked by the enriched proteasome-mediated ubiquitin-dependent protein catabolic process (GO:0043161). This arm was essential for maintaining proteostasis under inflammatory stress, clearing damaged proteins, and dynamically regulating immune signaling pathways (through targeted degradation). Additional enrichments in protein maturation by iron-sulfur cluster transfer (GO:0108035) and histone acetylation (GO:0016573) suggested concomitant investments in the functional activation of nascent proteins and epigenetic reprogramming that drive the transcriptional response. The co-dominance of these anabolic and catabolic programs effectively illustrated the profound proteostatic remodeling and resource reallocation central to the host defense.

CC analysis outlined a profound spatial reorganization and factory-scale expansion of the cellular architecture. By mapping the functional blueprint to the physical cellular architecture, we revealed a restructuring aimed at amplifying specialized biosynthetic and degradative factories (Figure 4B). The most insightful finding was the orchestrated enrichment of complexes governing the assembly and regulation of the ribosome and proteasome systems. Ribosome biogenesis involves key intermediates such as the Pwp2p-containing subcomplex of the 90S preribosome (GO:0030687) and the small-subunit processome (GO:0032040), signifying an active scaling of the assembly line. The enrichment associated with proteasomal degradation extended beyond the core proteasome complex (GO:0000502) to its regulatory apparatus (e.g., the proteasome regulatory particle (GO:0005838) and lid subcomplex (GO:0008540)), indicating fine-tuned degradation specificity and efficiency. This factory-scale expansion occurred within a broader cellular context with enriched fundamental compartments, including the nucleus (GO:0005634) and nucleolus (GO:0005730) for transcription and ribosome assembly and the cytosol (GO:0005829) for translation and proteolysis. Further extention of the adaptation was indicated by enrichments in structures like the centrosome (GO:0005813, potentially involved in cell division or immune synapse formation), exocyst (GO:0000145, involved in polarized exocytosis), and macropinosome (GO:0000415, involved in bulk endocytic uptake), along with machinery for mitochondrial biogenesis (mitochondrial intermembrane space protein transporter complex, GO:0042720) and protein modification and sorting (NatB complex, GO:0006465; Signal Recognition Particle, GO:1990357). These results demonstrated a system-wide spatial reorganization mobilizing the entire cellular infrastructure.

MF analysis defined the upgraded mechanistic toolkit enabling the above response. It detailed the specific biochemical activities comprising the upgraded infrastructure (Figure 4C). A massive bioenergetic investment occupied its foundation, evidenced by robust enrichment for ATP binding (GO:0005524) and ATPase activity, coupled with small GTPase binding (GO:0019003) for signal coordination. The prominent theme was the comprehensive upregulation of RNA-centric activities essential for post-transcriptional control, including RNA binding (GO:0003723), mRNA binding (GO:0003729), recognition of the RNA 7-methylguanosine cap (GO:0000339), and helicase activities (GO:0004386, GO:0003678). These were complemented by nuclear export signal receptor activity (GO:0008139) and ribonucleoprotein complex binding (GO:0043021), ensuring efficient mRNA processing and nucleocytoplasmic transport to feed the translational machinery. At the translational level, the enriched translation initiation factor activity (GO:0003743) directly supported boosted synthesis capacity, while aminoacyl-tRNA editing (GO:0006436) and glutamine-tRNA ligase activity (GO:0004816) safeguarded fidelity. The catabolic arm was pinpointed by ubiquitin-protein transferase activity (GO:0004842), identifying the enzymatic drivers of targeted degradation. Finally, enrichments in activities such as peptide alpha-N-acetyltransferase (GO:0008080) and histone acetyltransferase (GO:0004402) highlighted the rewiring of co- and post-translational modification landscapes.

Collectively, the integrated GO analyses revealed a host strategy across three interdependent levels: a strategic blueprint for prioritizing protein synthesis and turnover, a spatial reorganization to expand and optimize the physical factories that execute this blueprint, and a molecular toolkit upgrade that provides the necessary energy, specificity, and catalytic power. This tripartite adaptation underscores a profound commitment to resource reallocation and cellular infrastructure optimization, a core defense mechanism against *T. gondii* [34].

#### 3.3.3. PPI Network Analysis Identifies the Ribosome Biogenesis Machinery as the Topological Core

**Figure 5:**
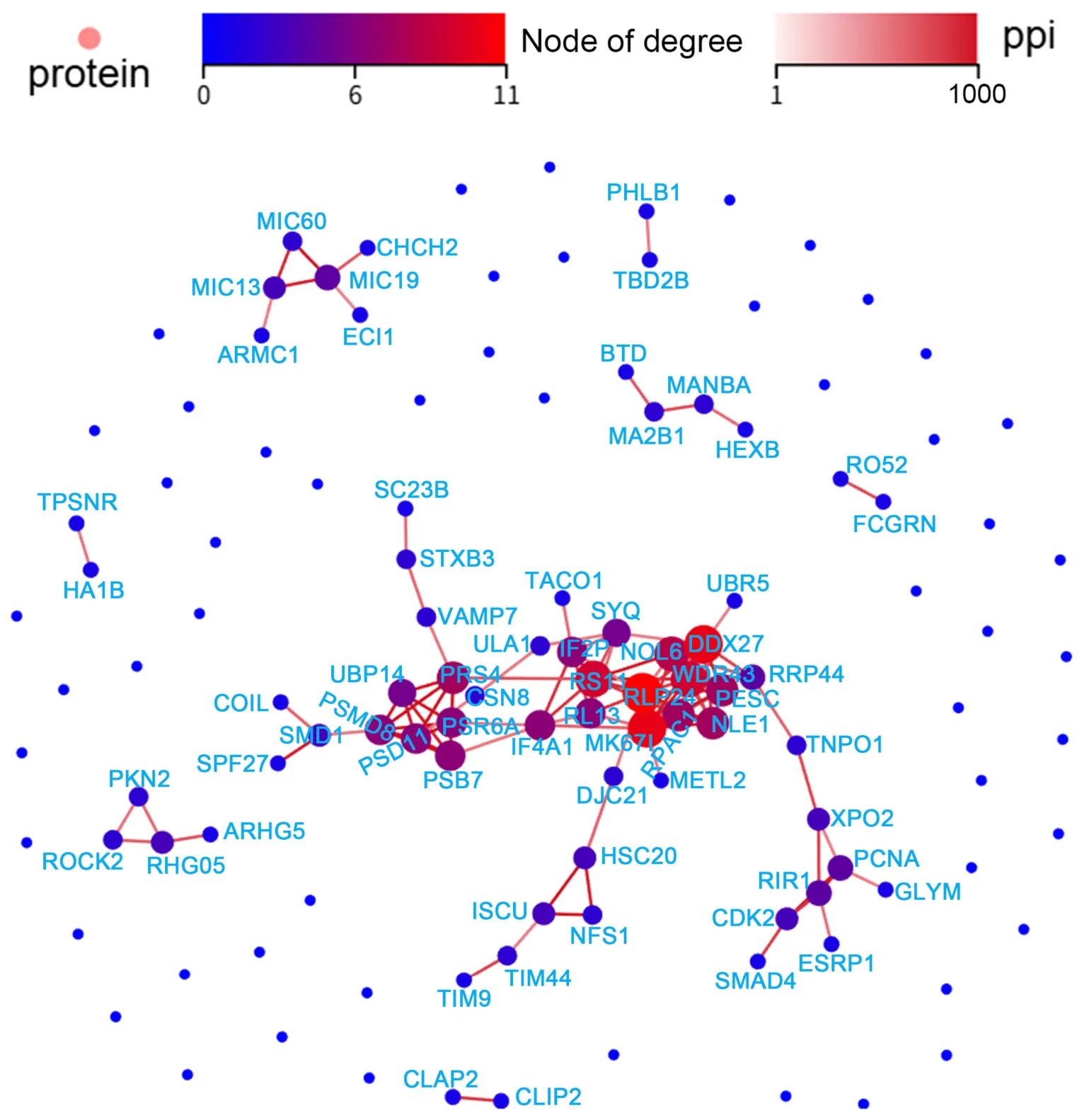
PPI network analysis of the host intestinal proteomic response. The PPI network was constructed based on the DEPs and the STRING database (confidence score > 700) and visualized using the Dr. Tom platform. Node color gradient (blue to red) corresponds to degree of centrality, with red nodes indicating high-connectivity hub proteins. Edge color reflects interaction confidence. The densely connected central cluster (red/purple nodes) is enriched for core hub proteins involved in ribosome biogenesis and RNA metabolism (e.g., DDX27, WDR43, and NOL6). Key functional modules related to protein ubiquitination (e.g., UBR5) and mitochondrial organization are also embedded within the network, reflecting coordinated regulation of translation, proteostasis, and metabolism during infection.

The systemic architecture governing the host intestinal response was elucidated by establishing the PPI network based on the DEPs and the STRING database (confidence score > 0.7), which was visualized using the Dr. Tom platform (BGI Genomics, Shenzhen, China) (Figure 5). The network exhibited a scale-free topology with high interconnectivity, i.e., the response is coordinated through functional modules rather than isolated events. Topological analysis identified the hub critical proteins for network integrity, marked by a red color gradient. Among them, key hubs included DEAD-box helicase 27 (DDX27), WD repeat-containing protein 43 (WDR43), and RNA polymerase I and III subunit C1 (RPAC1), all of which were centrally involved in ribosomal RNA processing and transcription. Their prominence underlines the fundamental role of the enhanced RNA processing and ribosome biogenesis machinery as a structural backbone for the global response. Module analysis using MCODE further decomposed the network into densely interconnected functional clusters. The most significantly enriched module was for ribosome biogenesis and translation initiation factors, entailing hubs such as DDX27 and WDR43, along with core components like eukaryotic translation initiation factors IF4A1 and IF2P. This module directly constitutes the physical interactome executing the upregulated anabolic pathway identified in prior analyses. The ubiquitin-proteasome system was represented by a distinct, high-scoring module, featuring regulatory particles (PSMD8 and PSD11) and catalytic core subunits (PSB7 and PRS6A). These results detail the molecular apparatus for the targeted protein catabolism that is essential for proteostasis and immune regulation. Additional modules included proteins implicated in cell cycle regulation (e.g., MK67I/Ki-67) and protein complex assembly, reflecting concomitant cellular processes driving the primary defense programs. Taken together, the host’s anti-parasitic strategy revolves around a tightly integrated core dedicated to protein synthesis capacity expansion, which is functionally and physically coupled with a dedicated protein degradation and remodeling apparatus [35]. This systems-level overview suggests that the host orchestrates its defense by reprogramming its functional interactome, prioritizing the reinforcement of the specific cellular infrastructure for protein production and turnover, thereby sustaining a potent and coordinated immune response.

Our study focuses on the proteomic characterization of the intestinal response at day 10 post-infection. Within this defined scope, the multi-level proteomic data are collectively consistent with a ‘defense-priority resource reallocation’ model, which is grounded in three convergent lines of evidence: (i) massive upregulation of immune effectors (84.1% of all DEPs); (ii) coordinated expansion of ribosome biogenesis and proteasome infrastructure at both KEGG and GO levels; and (iii) simultaneous downregulation of intestinal barrier components and digestive functions. These coexisting patterns argue for a regulated cellular resource reallocation from homeostatic maintenance to immune defense, as further substantiated by the GO tripartite architecture and PPI network topology. While the dataset herein does not distinguish whether this reallocation is host-driven or parasite-exploited, nor does it resolve whether the observed pathway enrichments reflect cell-autonomous reprogramming of epithelial cells or the increased representation of infiltrating immune cells, the coordinated nature of the observed changes suggests a regulated response rather than a passive collapse of intestinal function. Future studies employing cell-type-specific or spatial proteomics are essential for deconvoluting the contributions of distinct intestinal lineages to this coordinated response. We also regard integrating transcriptomics as a valuable future direction, which will require specifically designed multi-omics experiments with matched samples and time courses [36,37].

## 4. Conclusions

This study provides the first comprehensive quantitative map of intestinal proteome dynamics during acute oral *T. gondii* infection, revealing a profound and specific host proteome remodeling. The host response is dominated by an inflammatory and interferon-driven defense program, accompanied by compromised barrier integrity and suppressed digestive/absorptive functions. Mechanistically, the host strategically reinforces its protein synthesis and degradation machinery to sustain the mass production of immune effectors, reflecting a coordinated cellular infrastructure enhancement. These findings point to a novel paradigm of “defense-prioritized resource reallocation,” wherein energy and biosynthetic precursors are diverted from homeostatic functions to support rapid anti-parasitic immunity, at the cost of tissue pathology. This protein-centric resource reallocation moves beyond transcriptional correlations, highlighting critical post-transcriptional regulatory nodes and providing a systems-level framework for future investigations. The identified hub proteins and core modules represent promising targets for host-directed therapies. Based on the coordinated upregulation of proteasome and ribosome biogenesis pathways, for instance, pharmacological modulation of protein homeostasis, such as proteasome inhibitors or ribosome biogenesis modulators, could prove a rational strategy to interfere with the host cellular infrastructure that supports parasite replication and immune evasion. A framework is established for future preclinical evaluation of such host-directed interventions in the context of intestinal toxoplasmosis. Subsequent studies may focus on the temporal progression and cell type-specific contributions to fully decipher this coordinated host response.

## Data Availability

The mass spectrometry proteomics data have been deposited to the ProteomeXchange Consortium (https://www.iprox.cn/page/home.html, accessed on 17 August 2026) via the iProX partner repository [38] with the dataset identifier PXD082376.

## Supplementary Materials

The following supporting information can be downloaded at: https://www.mdpi.com/article/10.3390/biology15171461/s1, Table S1: Complete List of Identified Proteins; Table S2: Differentially Expressed Proteins.
